# Comparison of Random Forest and Gradient Boosting Machine Models for Predicting Demolition Waste Based on Small Datasets and Categorical Variables

**DOI:** 10.3390/ijerph18168530

**Published:** 2021-08-12

**Authors:** Gi-Wook Cha, Hyeun-Jun Moon, Young-Chan Kim

**Affiliations:** 1 Department of Architectural Engineering, Dankook University, Yongin 16890, Korea; cgwgnr@gmail.com; 2 Department of Safety Engineering, Dongguk University—Gyeongju, Gyeongju 38066, Korea; yyoungchani@gmail.com

**Keywords:** waste management, demolition waste, predictive model, bagging technique, boosting technique

## Abstract

Construction and demolition waste (DW) generation information has been recognized as a tool for providing useful information for waste management. Recently, numerous researchers have actively utilized artificial intelligence technology to establish accurate waste generation information. This study investigated the development of machine learning predictive models that can achieve predictive performance on small datasets composed of categorical variables. To this end, the random forest (RF) and gradient boosting machine (GBM) algorithms were adopted. To develop the models, 690 building datasets were established using data preprocessing and standardization. Hyperparameter tuning was performed to develop the RF and GBM models. The model performances were evaluated using the leave-one-out cross-validation technique. The study demonstrated that, for small datasets comprising mainly categorical variables, the bagging technique (RF) predictions were more stable and accurate than those of the boosting technique (GBM). However, GBM models demonstrated excellent predictive performance in some DW predictive models. Furthermore, the RF and GBM predictive models demonstrated significantly differing performance across different types of DW. Certain RF and GBM models demonstrated relatively low predictive performance. However, the remaining predictive models all demonstrated excellent predictive performance at *R*^2^ values > 0.6, and *R* values > 0.8. Such differences are mainly because of the characteristics of features applied to model development; we expect the application of additional features to improve the performance of the predictive models. The 11 DW predictive models developed in this study will be useful for establishing detailed DW management strategies.

## 1. Introduction

Waste management has become a critical issue due to rapid urban growth [1,2]. According to recent statistics, 2.01 billion tons of municipal solid waste (MSW) was generated in 2016; reports also predict that 3.40 billion tons of waste will be generated annually by 2050 [3]. In particular, construction and demolition (C&D) waste generation is steadily increasing [4,5,6], and 70–90% of C&D waste generation can be attributed to demolition activities [7,8]. For example, construction activities are responsible for 90% of the C&D waste generation in the United States; moreover, such activities account for 74% of the C&D waste generation in China [6,7,8]. C&D waste is mainly generated from such activities as construction, demolition, and refurbishment, which are detrimental to the environment because they generate considerable quantities of waste and greenhouse gases [9]. Therefore, systems or solutions for effectively and appropriately managing C&D waste are necessary for sustainable growth and development in the construction industry.

A key requirement for sustainable growth in C&D-related industries is to achieve the maximum economic and environmental values expected during building demolition [10]. To this end, accurate information on the amount of waste generation is necessary, as basic data for predicting the bulk of waste, the economic scale, and the environmental impact [6,11]. Therefore, information on the amount of C&D waste generation is perceived to be useful in informing the management of waste for relevant industry personnel (clients, architects, engineers, contractors, planners, etc.) [12].

Predicting C&D waste is a difficult task because it depends on the technical, cultural, and geometric variables of buildings. ML can be useful for explaining or understanding these variables by combining them; as such, its use in the construction industry has continued to increase [13]. Therefore, recently, numerous researchers have used artificial intelligence (AI) technology to establish accurate waste generation information; the unique characteristics of AI algorithms (data input, learning, and prediction) are considered state-of-the-art models for reliable waste generation prediction [14]. Various AI algorithms—including artificial neural networks (ANN), adaptive neuro-fuzzy inference systems (ANFIS), support vector machines (SVM), linear regression analysis (LR), and decision trees (DT)—have been applied as machine learning (ML) methods for the prediction of MSW and C&D waste generation. However, application of such AI systems requires *big data*, and insufficient data can be a major obstacle to the predictive performance of AI models [15]. Most existing studies, using such methods as ANN [10,16,17], SVM [16,18,19], and LR [16,20,21,22], are based on big data consisting of continuous variables; in other data environments (categorical variables, small datasets, etc.), the waste prediction performance seen in existing studies cannot be guaranteed [23], because predictive models using ML algorithms based on small datasets cannot be free of statistical bias [24] and high variance [25]. To overcome this limitation of applying AI systems, predictive models must be developed by applying ML algorithms suitable for small datasets composed of categorical variables. A recent study [26] demonstrated that DT-based algorithms exhibit excellent predictive performance for small datasets composed of categorical variables. Such results are expected to help DT-based ML algorithms improve the predictive performance of models for small datasets composed of categorical variables.

This study investigated the performance of predictive models of demolition waste (DW) generation by applying representative DT-based ML algorithms (RF and GBM) as a method to improve the predictive performance of AI models for small datasets composed of categorical variables. To this end, this study (1) built a dataset of information on the amount of generation of 10 types of DW, including the structure, region, purpose of use, wall materials, roof materials, and area variables. (2) To improve the performance of the predictive models, preprocessing was performed by eliminating outliers and normalizing the raw data. (3) Hyperparameters for each algorithm were tuned, and based on (1) and (2), GBM (boosting technique) and RF (bagging technique) algorithms—representative ensemble models based on DT—were applied to derive 10 types of waste generation models and one predictive model for total waste generation, covering all waste types. (4) Considering the characteristics of the data, leave-one-out cross-validation (LOOCV) was applied to verify and evaluate the predictive models. The performance of the models was evaluated using Pearson’s correlation coefficient (*R*), root mean square error (*RMSE*), coefficient of determination (*R*^2^), and mean absolute error (*MAE*) metrics. (5) Finally, the ensemble technique suitable for small datasets composed mainly of categorical variables was discussed by comparatively analyzing the predictive performance of the boosting technique and the bagging technique. This study sought to propose ensemble models that effectively improve the performance of predictive models for small datasets consisting mainly of categorical variables, and to discuss further research directions for improving predictive performance in limited data environments.

## 2. Description of Artificial Intelligence in Predicting DW Generation in This Study

In this section, we explore the principles and properties of random forest (RF) and gradient boosting machine (GBM) algorithms along with the properties of ensembles. Moreover, we explore LOOCV techniques applied for the performance evaluation of models based on small datasets mainly comprising categorical variables used in this study.

### 2.1. Ensemble Model

**Figure 1:**
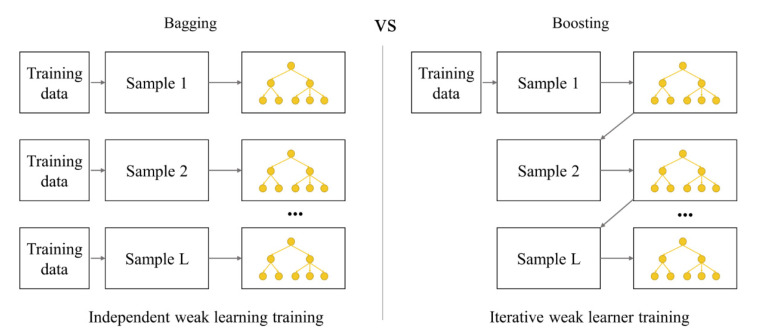
Workflows of bagging and boosting methods.

Ensemble learning (EL) methods involve combining and building various learning algorithms. This allows ensemble learning algorithms (ELA) to obtain better predictive performance and improved generalization compared to a single learning algorithm [27,28]. Ensemble methods are especially useful when the amount of training data is small. This is because ensemble algorithms (EAs) can reduce the risk of selecting a poor classifier through votes by individual classifiers [29]. Various ensemble techniques have been developed so far, among which bagging and boosting are representative of decision tree (DT)-based ELAs [30,31]. As presented in Figure 1, bagging (also known as bootstrap aggregation [32]) generates numerous bootstraps from the given training data, and an independent predictive model is generated for each bootstrap. Thus, bagging can improve the stability and accuracy of machine learning algorithms (MLA) [33]. Recently, RF has been widely utilized as a bagging method for MLA; Cha et al. and Nguyen et al. [23,34] demonstrated high predictive performance in applying RF to predict waste generation. Boosting, on the other hand, is a technique in which numerous classifiers are generated from early samples, and weak classifiers are collected to generate strong classifiers. As seen in Figure 1, bagging is an independence-based learner training system, while boosting is an iterative and dependence-based system. Boosting is a continuous process of generating classifiers reinforced by weights from weak classifiers from previous stages, which helps reduce the bias and variance of datasets [32]. GBM is representative of boosting DT-based algorithms. Johnson et al. [35] and Kontokosta et al. [36] applied GBM to predict the amount of MSW generation.

### 2.2. Gradient Boosting Machine and Random Forest

**Figure 2:**
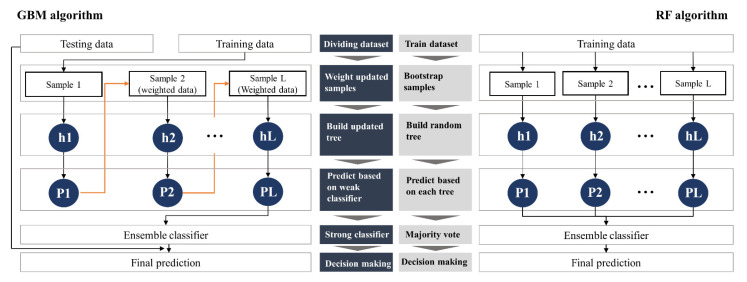
Comparison of the structure and workflows of the gradient boosting machine (GBM) and random forest (RF) algorithms.

GBM, as one of the most robust machine learning (ML) algorithms, is widely applied in engineering fields [37] and is a boosting technique. GBM may be deemed a numerical optimization algorithm aimed at finding an additive model that minimizes the loss function. To this end, GBM iteratively adds a new DT (*weak classifier* in Figure 2), which can reduce the loss function as much as possible at each stage. In other words, at each step, a new DT is fitted to the current residual and added to the previous model to update the residual. As presented in Figure 2, GBM is an iterative and dependence-based algorithm; as it iteratively performs these processes, it increasingly reinforces the classifier by the number of iterations specified by the user. GBM algorithms based on this boosting principle are effective in reducing bias and variance in predictive models [38]. Such characteristics of GBM are deemed useful in solving issues of bias and variance in predictive model results that occur when ML algorithms are applied to small datasets. Therefore, this study utilized GBM to predict demolition waste (DW) generation in a small-dataset environment consisting mainly of categorical variables.

RF is considered among the 10 best classifiers [39]. RF, a representative DT-based algorithm, is a bagging-based ensemble technique that generates bootstrap sampling (Figure 2). RF builds numerous subsets (bootstrap sampling) from the training data and trains the same algorithm several times. The resulting prediction is determined as the mean of all predictions of the submodels. As the number of trees increases, RF can avoid overfitting and be less affected by outliers. Moreover, even when the class is imbalanced, it has superior predictive power over other ML algorithms [33]. Considering these strengths, the application of RF in the data environment utilized in this study (small datasets composed mainly of categorical variables) is also expected to be useful for predicting DW generation.

### 2.3. Leave-One-Out Cross-Validation (LOOCV)

**Figure 3:**
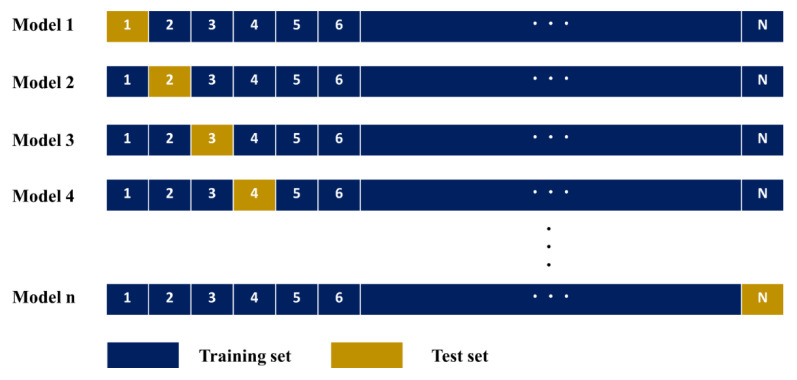
Schematic representation of the leave-one-out cross-validation (LOOCV) method.

K-fold and leave-one-out cross-validation (LOOCV) are widely used to evaluate the performance of classification algorithms. For large amounts of data, k-fold cross-validation (CV) should be applied to assess the accuracy of the classification model [40]. LOOCV is a special case of k-fold CV, in which the number of folds is equal to the number of instances (as shown in Figure 3). That is, LOOCV uses all samples in the dataset as the test and training data. The benefit of using so many fitted and evaluated models is a more robust estimate of model performance, as each row of data is given an opportunity to represent the entirety of the test dataset [23]. Therefore, when the number of instances in either a dataset or a class value is small, LOOCV must be applied to verify the accuracy of the classification algorithm [41]. These characteristics of LOOCV were considered in this study when selecting it as the CV method for the performance evaluation of the predictive models.

## 3. Materials and Methods

### 3.1. Data Source of Demolition Waste Generation (DWG) Data

This study utilized existing raw data [23,26,42] to develop predictive models for demolition waste (DW) generation. Raw data pertaining to the urban regeneration project districts in Daegu (35.88° N latitude, 128.61° E longitude) and Busan (35.87° N latitude, 128.63° E longitude) in the southern part of Korea were obtained. Therefore, the target buildings surveyed mainly included low-rise detached houses. Table 1 lists the statuses of the buildings whose raw data were used in this study.

Raw data were collected through investigations of the main members, areas, number of floors, structures, material types (i.e., mortar, concrete, block, brick, timber, slate, roofing tile, ceramic and glass, metal, or soil), characteristics, and sizes of the buildings prior to dismantling. These investigations were conducted using teams of two individuals, where one focused on measurements and the other focused on recording the data. The raw data included information on the amount of generation (kg/m^2^) of 10 types of DW (mortar, concrete, block, brick, timber, slate, roofing tile, ceramic and glass, metal, and soil) from 784 buildings. For each building, information is included on six types of construction characteristics (gross floor area, region, building structure, building use, wall material, and roofing material), which correspond to input variables that affect DW generation. The unit of DW generation data used in this study is kg/m^2^, in accordance with the following equation:

(1) $$DWGR_{i}\;of\;building_{k}=\frac{\sum A_{ij}\;of\;building}{GFA\;of\;building_{k}}$$

where *DWGR* is the demolition waste generation rate (kg/m^2^), $A_{ij}$ is the amount of waste material *j* with properties *i* (kg), and *GFA* is the gross floor area (m^2^) of building *k.*

### 3.2. Data Preprocessing and Preparing Datasets for Prediction Models

Preprocessing data are necessary to reduce the impact of data distortion or outliers and include cutting, adding, and transforming training datasets [43,44]. A stable dataset construction is required to improve the performance of the predictive model. Therefore, this study performed data outlier elimination and standardization of data to reduce the impact of data distortion and outliers resulting from the large variation in the collected raw data. In this study, the interquartile range method was used to eliminate outliers in the screening of data for use in the models, in accordance with Equation (2). After outlier removal, 690 out of the 784 building samples were used to develop the RF and GBM predictive models in this study.

Q1 − 1.5 × IQR < selecting data < Q3 + 1.5 × IQR, (2)

where IQR is the interquartile range and the value of IQR is Q3 minus Q1, Q1 being the 25th percentile, and Q3 the 75th percentile (Q represents quartile).

Standardization was performed to reduce the impact of data outliers on model performance and to build datasets of the same scales and units. Data standardization was conducted using Equation (3), in which the average of the data was $x_{average}$ and the standard deviation was $\sigma_{standard\;deviation}$.

(3) $$x_{standardization}=\frac{x_{element}-x_{average}}{\sigma_{standard\;deviation}}$$

### 3.3. Characteristics and Composition of Variables

Table 2 presents explanations of the data used in this study. Six independent variables and one dependent variable (DW generation) were used to develop the RF and GBM predictive models. The independent variables consist of nominal variables (region, use, structure, wall material, and roofing material) and one numeric variable (*GFA*). As shown in Table 2, the values converted into scales were utilized as input variables for the nominal variables.

### 3.4. Application of Machine Learning Techniques

The RF and GBM algorithms were selected to develop predictive models for DW generation for datasets with small samples and were composed mainly of categorical variables. In this study, RF and GBM algorithms were applied to develop 10 models for 10 types of DW, and one model for total DW generation including all 10 types of DW. The RandomForestClassifier [45] and GradientBoostingClassifier [46] packages were used to develop predictive models for DW generation. For optimal performance and evaluation upon application of the algorithm, parameters were tuned for the RF and GBM algorithms, including *n_estimators* (the number of trees to be grown). The parameters were tuned to maximize each model’s prediction accuracy on the dataset. The optimal settings were determined using the LOOCV process. Explanations of the hyperparameters applied to develop the RF and GBM models in this study are presented in Table 3.

### 3.5. Model Validation

The LOOCV validation technique was used to validate the models. Because LOOCV puts all samples through tests, it has the advantage of achieving stable results when targeting small datasets compared to the validation set approach, which is an existing cross-validation method (10-fold or k-fold) [23,47]. Several techniques that can be used for model performance evaluation are available. In this study, four statistical metrics (*MAE*, *RMSE*, *R*^2^, and *R*) were utilized to verify the performance of the models. Definitions of the performance evaluation metrics are presented in Equations (4)–(7).

(4) $$\mathrm{MAE}=\frac{\sum_{i=1}^{n}\left|y_{i}-x_{i}\right|}{n}$$

(5) $$\mathrm{RMSE}=\sqrt{\sum_{i=1}^{n}\frac{{\left(y_{i}-x_{i}\right)}^{2}}{n}}$$

(6) $$R^{2}=1-\frac{\sum_{i=1}^{n}{\left(y_{i}-x_{i}\right)}^{2}}{\sum_{i=1}^{n}{\left(y_{i}-{\bar{x}}_{i}\right)}^{2}}$$

(7) $$R=\frac{\sum_{i=1}^{n}\left(x_{i}-{\bar{x}}_{i}\right)\left(y_{i}-{\bar{y}}_{i}\right)}{\sqrt{\sum_{i=1}^{n}{\left(x_{i}-{\bar{x}}_{i}\right)}^{2}}\sqrt{\sum_{i=1}^{n}{\left(y_{i}-{\bar{y}}_{i}\right)}^{2}}}$$

where $x_{i}$ is the observed value of the generated DW amount, $y_{i}$ is the predicted value of the generated DW amount, ${\bar{x}}_{i}$ is the mean observed value of the generated DW amounts, ${\bar{y}}_{i}$ is the mean predicted value of the generated DW amount, and *n* is the number of samples.

## 4. Results and Discussions

### 4.1. Model Performance

**Figure 4:**
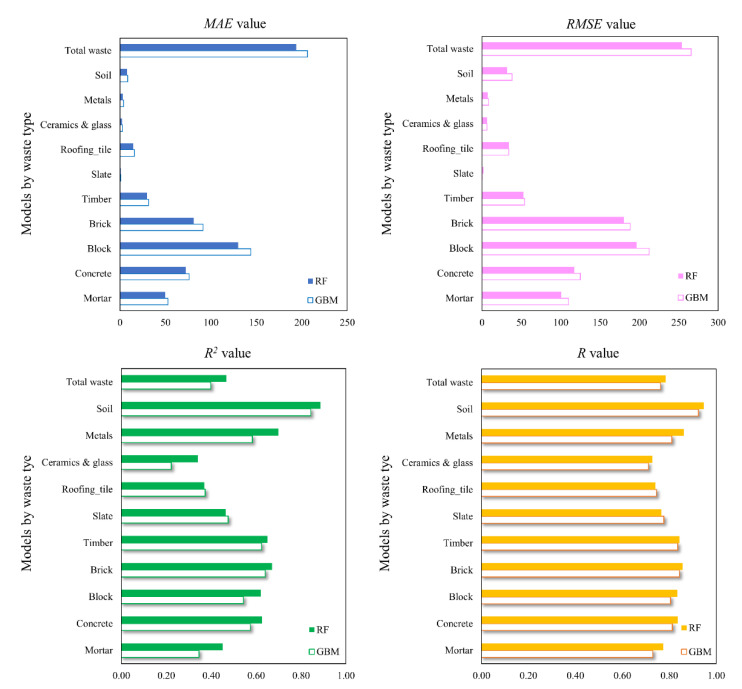
Comparison of statistical metrics (*MAE*, *RMSE*, *R*^2^, *R*) for GBM and RF models developed in this study. *MAE*, mean absolute error; *RMSE*, root mean square error; *R*^2^, coefficient of determination; *R*, Pearson’s correlation coefficient.

Figure 4 presents the comparison results of the performance metrics in the 11 predictive models for DW. According to the results of *MAE* and *RMSE* values in Figure 4, which indicate the stability of predictive performance, the *MAE* values of the RF models in all 11 predictive models are lower than those of the GBM models. *MAE* values are slightly lower in the 10 predictive models for each DW, and also in the total DW production predictive model: the *MAE* value of the RF model (*MAE*: 193.7) is lower than that of the GBM model (*MAE*: 206.2). According to the *RMSE* results, the GBM models (*RMSE* value of slate is 0.20, and *RMSE* value of roofing tile is 34.02) for the slate and roofing tile DW predictive models indicate slightly more stable predictive performance than achieved by the RF model (*RMSE* value of slate is 2.04, and *RMSE* value of roofing tile is 34.31). However, the *RMSE* values of the remaining DW predictive models are lower in the RF model than in the GBM model. The *MAE* and *RMSE* results demonstrate that, in the predictive models for small datasets of categorical variables, RF algorithms generally have slightly more reliable predictive performance than achieved by GBM algorithms. In other words, the bagging technique appears to have a slightly better predictive performance in terms of stability over the boosting technique.

**Figure 5:**
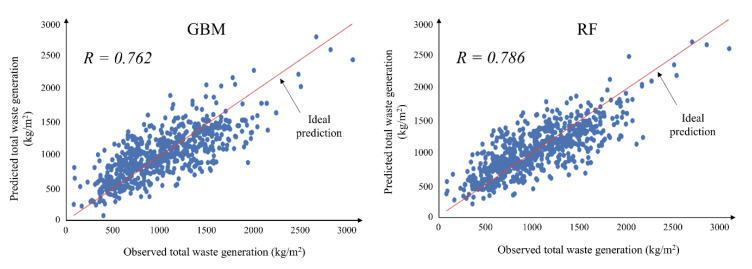
Scatter plot of the observed and predicted total DW generation rate including all waste types using GBM and RF.

Figure 4 shows for each type of DW that RF predictive models (*R*^2^ values: 0.34–0.89; *R* values: 0.73–0.95) are generally superior in terms of the accuracy of predictive performance compared with GBM predictive models (*R*^2^ values: 0.22–0.84; R values: 0.71–0.92). Furthermore, from Figure 5, it can be seen that the scatterplots of predicted and observed values of the total DW predictive model conform more closely to ideal prediction lines in the RF model than in the GBM model. However, GBM models demonstrate slightly better predictive performance in terms of accuracy: the *R*^2^ and *R* values for slate are 0.48 and 0.78, respectively, in the GBM model, and 0.46 and 0.77 in the RF model; *R*^2^ and *R* values for roofing tiles are 0.37 and 0.75, respectively, in the GBM model, and 0.37 and 0.74 in the RF model. Considering the models overall, the RF models are slightly superior in terms of the accuracy of DW predictive models, and it is judged that the bagging technique will be more useful than the boosting technique for developing accurate models for small datasets composed of categorical variables.

Meanwhile, the predictive performance based on the type of DW showed different results. This difference can be attributed to the characteristics of the features applied to the model development. In other words, it is expected that more stable performance results can be obtained if the variables affecting waste generation with respect to waste type are included, apart from the six variables used in this study. For instance, the features used in this study (i.e., region, use, structure, *GFA*, WM, and RM) are considered to be appropriate key features as input variables affecting the amount of soil generation. On the other hand, the ceramic and glass predictive model with the lowest prediction performance requires additional input variables (such as the external window area ratio), apart from the six variables used in this study. Similar findings were also reported for MSW by Johnson et al. [34], who applied the GBM algorithm to apply feature compositions differently and developed predictive models for refuse, paper, and MGP (metal, glass, and plastic). Each predictive model developed in that study showed different *R*^2^ and *RMSE* results depending on the external or internal characteristics of the applied features.

### 4.2. Comparison of Predictive Models

Although all RF and GBM models developed in this study were derived from a small dataset, the correlation between the actual and predicted values (*R* value) is 0.7 or higher, demonstrating excellent predictive performance (Figure 4). However, while some DWs (mortar, roofing tile, ceramic, and glass) have slightly lower *R*^2^ values (0.4 or lower), their *R* values are all 0.7 or higher, and thus no issues are expected for the accuracy of the predictive performance. However, these results suggest that it is necessary to include key features considering the characteristics of buildings that affect the DWG for DW types with low predictive performance. In addition, it is expected that this will help improve the *R*^2^ value, because this value gradually increases as new variables are introduced into the model [48,49].

**Figure 6:**
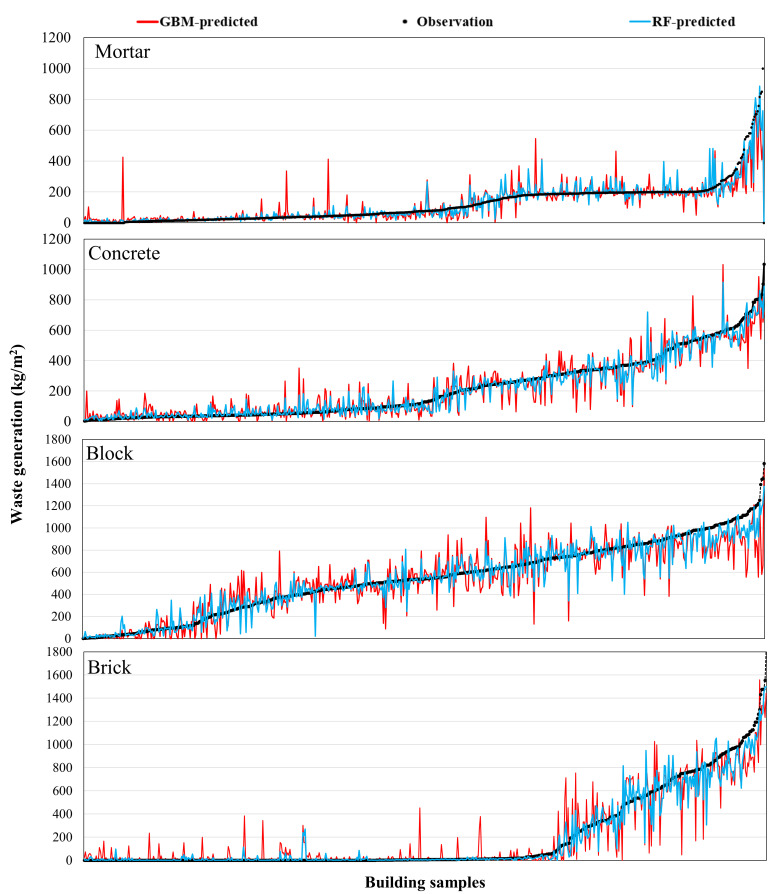
Comparison of the observed and predicted DW generation (kg/m^2^) with the estimated ML models using RF and GBM for mortar, concrete, block, and brick.

**Figure 7:**
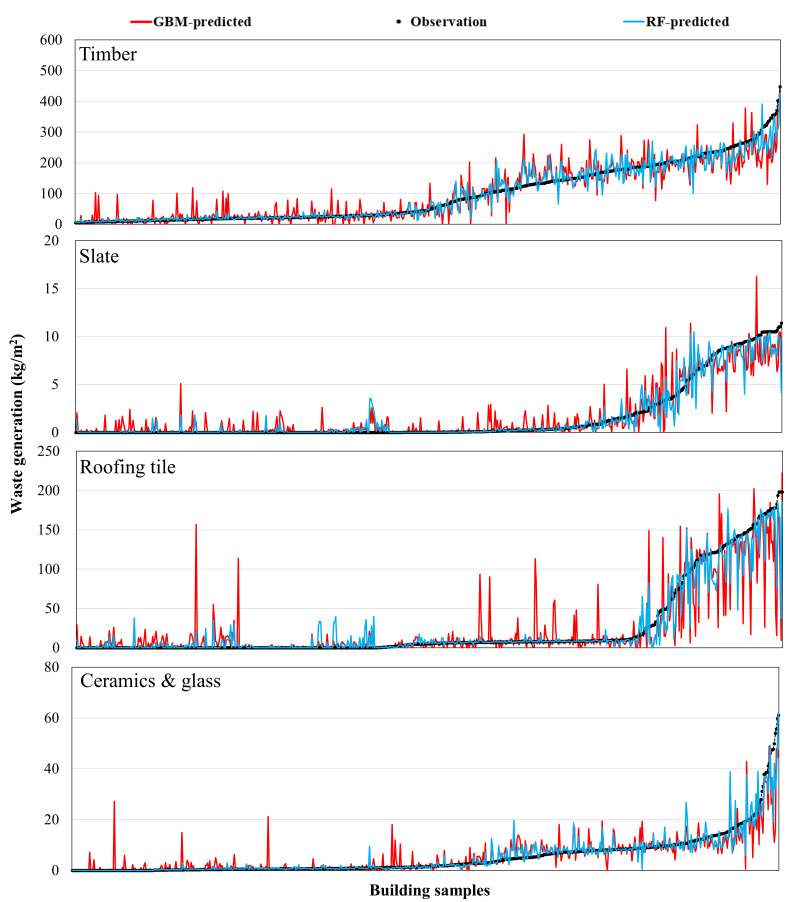
Comparison of the observed and predicted DW generation (kg/m^2^) with the estimated ML models using RF and GBM for timber, slate, roofing tile, and ceramics & glass.

**Figure 8:**
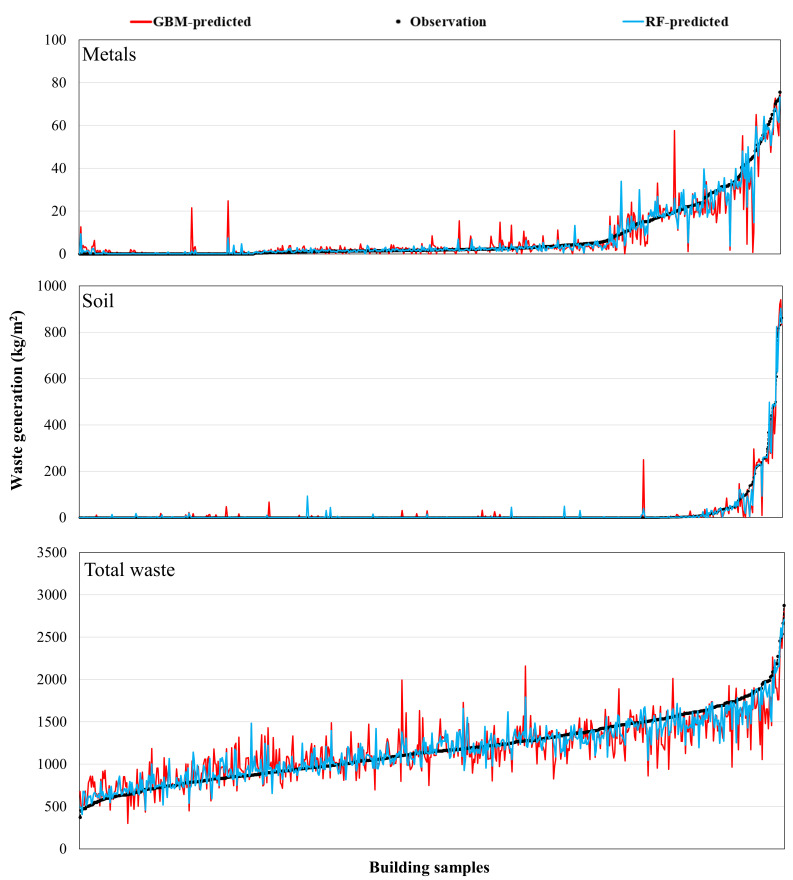
Comparison of the observed and predicted DW generation (kg/m^2^) with the estimated ML models using RF and GBM for metal, soil, and total waste.

Figure 6, Figure 7 and Figure 8 present the comparison results of the observed and predicted DW models using the RF and GMB algorithms; the results for all 11 RF and GBM models indicate that the actual DW generation patterns are well simulated. The mean observed value of the soil predictive model, which had the best predictive performance, is 21.8 kg/m^2^, while the mean predicted value of the RF model (*R* value: 0.947) is 21.6 kg/m^2^, and that of the GBM model (*R* value: 0.824) is 21.4 kg/m^2^. The mean observed value of the ceramic and glass predictive model, which had the lowest predictive performance among DW predictive models, is 5.3 kg/ m^2^, where the mean predicted value of the RF model (*R* value: 0.729) is 5.4 kg/m^2^, and that of the GBM model (*R* value: 0.711) is 5.5 kg/m^2^. The mean observed value of the total waste predictive model is 1171.2 kg/m^2^, while the mean predicted value of the RF model (*R* value: 0.786) is 1169.9 kg/m^2^, and that of the GBM model (*R* value: 0.762) is 1166.4 kg/m^2^. Other predictive models showed results in which the predicted values of the RF and GBM models were close to the observed values. However, as seen in the *MAE* and *RMSE* results in Figure 4, the GBM models produced some predicted values that were high compared with values produced by the RF models, indicating that stable predictive performance is somewhat poor (Figure 6, Figure 7 and Figure 8).

### 4.3. Discussion, Limitations, and Future Work

AI models in previous studies [19,35,50] presented results for some DW types. Johnson et al. [35] applied GBM algorithms to conduct research on refuse, paper, and MGP (metal, glass, and plastic) predictive models. In this study, *R*^2^ results for refuse, paper, and MGP spatial models, to which features of external groups were applied, demonstrated predictive performances of 0.604, 0.628, and 0.428, respectively. The predictive performance of MSW and paper predictive models, to which decision tree (DT) algorithms were applied in the study by Kannangara et al. [50], was 0.54 and 0.31, respectively. In the study by Kumar et al. [19], the *R*^2^ of the plastic generation predictive model, to which the RF algorithm was applied, demonstrated a predictive performance of 0.66. This study proposes predictive models for the generation of 10 types of DW, and one for total waste generation, for small datasets. In this study, the 11 RF and GBM models developed, despite targeting small datasets, demonstrated excellent predictive performance with the correlation (*R*) of the observed and predicted values at 0.73–0.95 (RF models) and 0.71–0.92 (GBM models), respectively. *R*^2^ values also demonstrated excellent predictive performances of 0.6 or higher, except for mortar, slate, roofing tile, ceramics, and glass. However, as AI models depend on datasets of large sizes [15], a dataset size that is not sufficiently large is a fundamental limitation of this study; obtaining datasets that are sufficiently large is a significant challenge. This study also found significant differences in the performance (*MAE*, *RMSE*, *R*^2^, *R*) of predictive models depending on the type of DW, and the *R*^2^ values for demolition waste materials such as slate, mortar, roofing tile, ceramic, and glass were relatively low (≤0.5), demonstrating relatively low performance. Johnson et al. [35] suggested that the development of optimal feature groups is also important for improving the predictive performance of AI models by demonstrating that differences in the composition and characteristics of features applied to AI models affect the results of predictive performance. Based on this, it is deemed that further features must be developed to improve the performance of predictive models for the types of DW that had relatively low predictive performance in the present study.

## 5. Conclusions

This study investigated the development of AI models for DW generation suitable for small datasets composed mainly of categorical variables. The DT-based ensemble model was applied as an algorithm suitable for the datasets, with one selected algorithm being the RF algorithm, which is representative of the bagging technique, and the other being the GBM algorithm representative of the boosting technique. Data preprocessing was performed to improve the stability and accuracy of the models, and the parameters were tuned to fit the RF and GBM algorithms. The LOOCV technique was applied to verify the developed RF and GBM models, and performance evaluation was conducted using statistical metrics such as *MAE*, *RMSE*, *R*^2^, and *R*. Therefore, the consideration of AI models developed in this study for small datasets composed mainly of categorical variables, along with the RF and GBM predictive models developed for the generation of 10 types of DW and one for total DW generation, is summarized as follows.

First, for small datasets composed mainly of categorical variables, the bagging technique (RF model) was found to be superior to the boosting technique (GBM model) in terms of the stability and accuracy of predictive performance. However, the GBM model demonstrated excellent predictive performance for certain types of DW (slate and roofing tile). Therefore, the selection of appropriate RF and GBM algorithms, depending on the type of DW, is necessary in developing DW prediction models for small datasets composed mainly of categorical variables.

Second, 11 RF (*R*^2^ values: 0.22–0.84; *R* values: 0.71–0.92) and GBM (*R*^2^ values: 0.34–0.89; *R* values: 0.73–0.95) predictive models under identical conditions demonstrated differences in performance according to the type of DW. This is considered to be due to the characteristics of the features applied in developing the models. Therefore, it is expected that the performance of predictive models will be improved in the future if features matching the characteristics of DW with low predictive performance (mortar, slate, roofing tile, ceramic, and glass, having *R*^2^ values ≤0.5) are added.

Finally, it is recommended that RF methods be applied to develop DW predictive models for concrete, block, brick, timber, ceramic and glass, metals, soil, and total waste, while applying GBM models for slate and roofing tiles, based on 11 predictive models developed in this study. This study proposes predictive models for more types of DW than included in the AI models of previous studies. The results of this study are anticipated to be useful tools for building demolition businesses to establish thorough and detailed DW management strategies. For example, based on the predicted amount of waste to be generated from the type of DW, a demolition company can place orders for the required demolition equipment and transportation, which will be of use in minimizing excess costs and personnel allocations.

## Figures and Tables

**Table 1:** Building status of raw data used in this study.

Structure Type	Number of Buildings	Total Floor Area (m^2^)
RC	147	56,929
Masonry	352	33,291
Wood	285	22,750
Total	784	112,970

**Table 2:** Input and output variables used to develop the models in this study.

Variables Type	Variables	Description	Unit or Scale of Variables
Independent variables	Region	Nominal variable; Areas where DW has occurred; Three region variables (Region A, B, C)	Region A is 1 Region B is 2 Region C is 3
	Building use	Nominal variable; Usage of building where DW has occurred; Three usage variables (residential-only, commercial and residential, commercial-only)	Residential-only is 1 Commercial and Residential is 2 Commercial-only is 3
	Building structure	Nominal variable; Structure of building where DW has occurred; Three structure variables (reinforced concrete, masonry, wooden)	Reinforced concrete is 1 Masonry is 2 Wooden is 3
	Wall material	Nominal variable; Main wall material of building where DW has occurred; Four wall material variables (reinforced concrete wall, brick wall, block wall, wall made of soil)	Reinforced concrete wall is 1 Brick wall is 2 Block wall is 3 Wall made of soil is 4
	Roofing material	Nominal variable; Main roofing material of building where DW has occurred; Four roofing material variables (slab, slab and roofing tile, roof with asbestos, roofing tile)	Slab is 1 Slab and roofing tile is 2 Roof with asbestos is 3 Roofing tile is 4
	Gross floor area (*GFA*)	Numeric variable	m^2^
Dependent variable	Waste generation	Numeric variable	kg/m^2^

**Table 3:** Hyperparameters tuned in RF and GBM algorithms.

Algorithm	Parameter	Definition	Applied Value or Reference
RF	criterion	Quality measurement of a split	Mean squared error
	n_estimators	The number of trees in the forest	500
	min_samples_split	The minimum number of samples required to split an internal node	2
	min_samples_leaf	The minimum number of samples required to be at a leaf node	1
	max_depth	The maximum depth of the tree	Maximum possible
GBM	criterion	Quality measurement of a split	Mean squared error
	n_estimators	The number of boosting stages	500
	min_samples_split	The minimum number of samples required to split an internal node	2
	loss	Least squares	Least squares
	learning rate	Amount of learning	0.1
	subsample	Rate of sampling data to control overfitting	1.0

## Data Availability

The data that support the findings of this study are available from the corresponding author, upon reasonable request.
